# Supplementary material for: Endothelial-specific FoxO1 depletion prevents obesity-related disorders by increasing vascular metabolism and growth
Source: eLife. 2018 Dec 4;7:e39780. doi: 10.7554/eLife.39780 (PMC6279348; doi:10.7554/eLife.39780)
Supplement: Supplementary file 1. — Tissue weights of Control and EC-FoxO1,3 KD mice after 14 weeks of HF diet [file elife-39780-supp1.doc]

**Supplementary Table 1. Tissue weights of Control and EC-FoxO1,3 KD mice after 14 weeks of HF diet**

|  |  |  | **Control** | **EC-FoxO1,3 KD** |
| --- | --- | --- | --- | --- |
| Body weight (g) |  |  | 38.4 ± 0.8 | 34.6 ± 1.3* |
| eWAT (g) |  |  | 1.6 ± 0.1 | 1.5 ± 0.1 |
| rWAT (mg) |  |  | 985.5 ± 81 | 563.8 ± 66.1* |
| Subcutaneous WAT(g) |  |  | 1.5 ± 0.2 | 0.9 ± 0.1* |
| Liver (g) |  |  | 1.4 ± 0.1 | 1.4 ± 0.04 |
| Heart (mg) |  |  | 136.2 ± 3.4 | 123.9 ± 6.5 |
| Gastrocnemius (mg) |  |  | 122 ± 2.3 | 114 ± 2.5* |
| Soleus (mg) |  |  | 9.3 ± 0.6 | 9.9 ± 0.8 |
| Tibialis anterior (mg) |  |  | 45.2 ± 1.6 | 47.5 ± 2.3 |

eWAT: epididymal adipose tissue; rWAT: retroperitoneal adipose tissue

Data are expressed as mean ± S.E.M., n=6 per group

Significance was established using unpaired *t* test

* *P*<0.05 *vs* Control
