# Supplementary material for: Endothelial-specific FoxO1 depletion prevents obesity-related disorders by increasing vascular metabolism and growth
Source: eLife. 2018 Dec 4;7:e39780. doi: 10.7554/eLife.39780 (PMC6279348; doi:10.7554/eLife.39780)
Supplement: Supplementary file 2. [file elife-39780-supp2.doc]

**Supplementary Table 2 – TaqMan® primer sets**

| Gene | Probe ID  (ThermoFisher Scientific) |
| --- | --- |
| *Actb* | Mm04394036_g1 |
| *Adipoq* | Mm00456425_m1 |
| *Apln* | Mm00627688_g1 |
| *Ccnd1* | Mm00432359_m1 |
| *Cdkn1b* | Mm00438168_m1 |
| *Foxo1* | Mm00490671_m1 |
| *Foxo3a* | Mm01185722_m1 |
| *Hk2* | Mm00443385_m1 |
| *Hprt1* | Mm00446968_m1 |
| *Leptin* | Mm00434759_m1 |
| *Mki67* | Mm01278617_m1 |
| *Nos3* | Mm00435217_m1 |
| *Pecam1* | Mm00476712_m1 |
| *Pfkm* | Mm01309576_m1 |
| *Pfkfb3* | Mm00504642_m1 |
| *Prdm16* | Mm00712556_m1 |
| *Slc2a1* | Mm00441480_m1 |
| *Slc16a4* | Mm00525195_m1 |
| *Ucp1* | Mm01244861_m1 |
| *Vegfa* | Mm00437306_m1 |
| *Vwf* | Mm00550376_m1 |
